# Thermodynamic assessment of heat stress in dairy cattle: lessons from human biometeorology

**DOI:** 10.1007/s00484-022-02321-2

**Published:** 2022-07-11

**Authors:** Sepehr Foroushani, Thomas Amon

**Affiliations:** 1 Engineering for Livestock Management, Leibniz Institute for Agricultural Engineering and Bioeconomy, Potsdam, Germany; 2 Institute of Animal Hygiene and Environmental Health, College of Veterinary Medicine, Free University Berlin, Berlin, Germany

**Keywords:** Heat stress, Mechanistic model, Thermoregulation, Apparent temperature, Thermoregulatory exhaustion index

## Abstract

A versatile meteorological index for predicting heat stress in dairy cattle remains elusive. Despite numerous attempts at developing such indices and widespread use of some, there is growing skepticism about the accuracy and adequacy of the existing indices as well as the general statistical approach used to develop them. At the same time, precision farming of high-yielding animals in a drastically changing climate calls for more effective prediction and alleviation of heat stress. The present paper revisits classical work on human biometeorology, particularly the apparent temperature scale, to draw inspiration for advancing research on heat stress in dairy cattle. The importance of a detailed, mechanistic understanding of heat transfer and thermoregulation is demonstrated and reiterated. A model from the literature is used to construct a framework for identifying and characterizing conditions of potential heat stress. New parameters are proposed to translate the heat flux calculations based on heat-balance models into more tangible and more useful meteorological indices, including an apparent temperature for cattle and a thermoregulatory exhaustion index. A validation gap in the literature is identified as the main hindrance to the further development and deployment of heat-balance models. Recommendations are presented for systematically addressing this gap in particular and continuing research within the proposed framework in general.

## Introduction

### Overview

Heat stress in dairy cattle has been the subject of continued interest for several decades. A significant portion of the literature can be categorized as attempts to develop heat stress indices through regression analysis of meteorological parameters such as ambient temperature, humidity, wind speed, and solar radiation and animal responses such as body temperature, respiration rate, and milk yield, or select the “appropriate” index from the literature. In the latter case, meteorological data are used to evaluate various available indices, in search of indices that show significant statistical correlation with animal response data. Despite numerous attempts, a versatile index remains elusive as evidenced by continual revisions to existing indices and development of new indices. A recent review (Ji et al. 2020a) lists as many as 20 heat stress indices for dairy cattle.

An alternative, possibly complementary, approach is to use mechanistic models of heat generation and dissipation to identify conditions of potential stress based on the heat balance of the animal. Examples include the work of McArthur (1987), Ehrlemark and Sällvik (1996), Turnpenny et al. (2000a, b), McGovern and Bruce (2000), and Thompson et al. (2014). Despite its fundamental robustness, the heat-balance approach has attracted much less attention than the statistical approach and its application remains limited. Very few studies deal with the application and assessment of heat-balance models, including for instance the papers by Bloomberg and Bywater (2007) and van der Linden et al. (2018). Even fewer attempts have been made at systematic application of a heat-balance model to identify conditions of potential heat stress and to develop relevant indices accordingly. The work of Berman (2004, 2005, 2006) is a notable exception, which nevertheless also resorts to linear regression of modeling results to develop simplified indices, and in some cases, linear fits stretched beyond the range of the underlying empirical data. The relative unpopularity of mechanistic models can be attributed to their formal complexity, large number of input parameters and the need for iterative solutions as well as lack of experimental data that can be used for reliable estimation of the parameters or validation of the models, especially for the modern high-production dairy cow.

The present paper is an attempt to outline a path forward based on mechanistic models for predicting conditions of potential heat stress in dairy cattle. The many statistical indices are not reviewed here. The reader is instead referred to the recent publications by Ji et al. (2020a) and dos Santos et al. (2021). In the absence of similar reviews of heat-balance models, the few existing models are briefly discussed here. Adopting classical theoretical work on human thermal comfort and perception, particularly the apparent temperature scale (Steadman 1984), two basic questions are examined: first, is the hitherto shortcoming of the conventional (statistical) approach methodological? Second, is there inspiration to be drawn from human biometeorology? More specifically, how can thermodynamic modeling and prediction of heat stress in cattle offer new perspectives and possibilities?

### Thermoregulation

As homeotherms, cattle maintain a relatively constant core body temperature in a range known as the “thermoneutral” zone, where minimal energy is spent on thermoregulation and maximal energy is devoted to metabolism and production (Mount 1974; Godyń et al. 2019). Beyond this zone, thermoregulatory mechanisms are activated to return to the thermoneutral zone (dos Santos et al. 2021). There is a limit to the effectiveness of the thermoregulatory mechanisms beyond which thermal stress, hyperthermia or hypothermia, occurs (Kamal et al. 2016; Sejian et al. 2018). In this zone, behavioral changes such as reduced feed intake, increased water intake, and reduced lying time and seeking shade are triggered as secondary coping mechanisms, effectively assisting the physiological thermoregulatory responses (Ratnakaran et al. 2017; Madhusoodan et al. 2019). In thermodynamic terms, the response to heat stress can be divided into (1) increasing heat dissipation (total heat transfer coefficient), e.g. through enhanced perspiration and increased surface (skin) temperature, and (2) reducing the endogenous heat generation, e.g. by reducing feed intake and activity. Hyperthermia (heat stress) occurs when the heat dissipation cannot be adequately modulated to meet the thermoneutral heat generation and maintain the basal (normal) body temperature (Spiers 2012). For a detailed discussion of thermoregulation and thermal stress in cattle, see the review by dos Santos et al. (2021) and the sources therein cited.

### Heat stress: indicators and predictors

As pointed out by West (2003), the term heat stress is used rather loosely to signify the climate, climatic effects, or the animal’s response. Alternative terminologies are also used in the literature, e.g. “heat load” by Heinicke et al. (2018), with the same meanings. Here, the definition put forth by Lee (1965) is adopted where heat stress means “the conditions that displace the animal’s thermoregulation system out of the thermoneutral zone,” and heat strain is accordingly “the displacement or deviation of the physiological, behavioral, or productive parameters from the corresponding base values in the thermoneutral zone.”

Heat strains are “indicators” of heat stress, i.e. signs that heat stress is occurring (e.g. increased respiration rate) or has already occurred (e.g. reduced milk yield). Direct reliance on such indicators for environmental control in livestock management would be challenging as it requires close, real-time monitoring of various physiological parameters and operation of the environmental control systems based on such observations. Adding to this challenge is the fact that some thermoregulatory responses (indicators) such as sweating or breathing tend to be highly variable from animal to animal, even within the same genotype or herd. See for instance the work of Maia et al. (2005a, b) and Gebremedhin et al. (2010) on dairy cattle and similarly the work of Gaughan et al. (2010) on beef cattle. Meteorological parameters, on the other hand, are readily available either from on-farm measurements or nearby weather stations. Furthermore, some indicators can only be observed when stress has already occurred [milk yield reduction (dos Santos et al. 2021)] or is well underway (core body temperature rise). Therefore, indices based on meteorological parameters, primarily ambient temperature, humidity, and wind speed, are sought to identify and categorize the conditions that are likely to cause heat stress. In this sense, a heat stress index serves as a “predictor.”

Despite more than 60 years of research, the observation by Berman (2005) that no clear criteria exist for conditions in which heat stress relief is needed remains the case. Most existing heat stress indices are applicable to temporally averaged, herd-level indicators. Even at this resolution, correlations are highly variable. See, for instance, the correlations of daily milk yield and milk temperature with ten different environmental indices presented in the paper by Ji et al. (2020b). Such levels of resolution and accuracy can be inadequate to address the needs of precision livestock farming in a rapidly and drastically changing climate.

As shown in a recent review (Ji et al. 2020a), the 40 years following the introduction of the temperature-humidity index [THI (Thom and Bosen 1959; Bianca 1962)] can be roughly summarized as readjustments and reformulations of essentially the same index (eight variants of THI and several similar indices). The alternative indices proposed in the last 20 years, e.g. the Comprehensive Climate Index [CCI (Mader et al. 2010)], are more complex in form, but were derived following the same general methodology, i.e. starting with some variant of THI and introducing incremental regression-based adjustments for air speed or solar radiation. See, for example, the development of the Heat Load Index [HLI (Gaughan et al. 2008)] and its expansion to CCI (Mader et al. 2010). Moreover, some of the adjustments demonstrate non-physical features. For example, the wind speed adjustments in the high-radiation variant of HLI (Gaughan et al. 2008) and CCI (Mader et al. 2010) have non-zero intercepts (respectively -11°C and +3°C at *u*=0) and non-zero slopes even at wind speeds as high as 25 m/s, i.e. non-asymptotic behavior.^1^ Finally, the adjustments, particularly the wind-speed term in CCI (Mader et al. 2010), are rather complex in form.

More recently, indices such as the Dairy Heat Load Index [DHLI (Lees et al. 2018)] and the Equivalent Temperature Index for Cattle [ETIC (Wang et al. 2018)] have presented a breakaway from the practice of incremental improvements to THI, although they too rely almost exclusively on regression analyses of meteorological parameters and animal responses. A recent study (Ji et al. 2020b) has found the new indices (DHLI, ETIC) to predict heat stress no better than the older indices (THI, HLI, CCI). A more recent study (Lees et al. 2022) concluded the relative success of DHLI and THI in predicting heat stress depends on the physiological/behavioral indicator of interest (panting, drinking, standing) as well as the animals’ access to shade. The statistical indices discussed above consider the intensity of heat stress only. In other words, the duration of heat exposure (or relief) is not considered. Gaughan et al. (2008) have called this a “one-dimensional” approach. A few studies have attempted to incorporate the transient nature of the thermal interaction between animals and their surrounding, and specifically the effects of prolonged heat exposure and intermittent relief (e.g. at night). Relying on the classical THI index, Hahn and Mader (1997) proposed the hours above established THI thresholds (“THI-hours”) to be considered in the forecast of heat waves. Heinicke et al. (2018) have used a similar approach, based on THI and lying/standing behavior, to examine the effects of the duration of heat exposure in terms of a heat load duration (HLD) index. Similarly, Gaughan et al. (2008) used the length of the periods when the heat load index (HLI; see discussion in preceding paragraphs) is above or below a critical threshold to develop the Accumulated Heat Load (AHL) index. Methodologically, the AHL model offers notable advancement as it includes the effects of wind speed and solar radiation, which are absent from THI, as well as the effects of heat relief (HLI<threshold).

Although the premise of the “two-dimensional” heat load duration indices mentioned above is the consideration of heat balance (and heat accumulation in the case of bodily heat surplus), they are no different than the “one-dimensional" indices in their reliance on purely statistical correlations as a proxy for the thermal interaction between the animal and the environment. As pointed out by Ehrlemark and Sällvik (1996), it is a major shortcoming of the statistical approach that it ignores the thermodynamics of thermoregulation and heat dissipation. Ehrlemark and Sällvik (1996) found it therefore “not surprising” that practical experience from livestock management shows significant deviations from the predictions of the statistical models. Heat-balance models based on thermodynamic principles have the potential to address that shortcoming and complement statistical correlations between meteorological and physiological observations.

## Heat-balance models for cattle

Research on heat-balance models for livestock has a history of more than three decades. Finding earlier thermal models, e.g. Porter and Gates (1969), limited in their incorporation of the thermoregulatory responses, McArthur (1987) developed a detailed steady-state heat-balance model for homeothermic vertebrates, which entailed the physiological responses. Despite the formal simplicity of the basic equation, the submodels used by McArthur (1987) to describe the underlying physical and physiological phenomena are rather complicated, entailing a high degree of non-linearity and coupling. Following the same general approach, Ehrlemark and Sällvik (1996) developed a steady-state heat-balance model. The ANIBAL (ANImal heat BALance) model (Ehrlemark and Sällvik 1996) is much simpler than the model by McArthur (1987) with potentially greater utility. Nevertheless, ANIBAL was not used to identify conditions of potential heat stress, but rather to predict heat generation at low (ambient) temperatures and evaporative heat loss at high (ambient) temperatures (Ehrlemark and Sällvik 1996). Moreover, while criticizing the traditional statistical approach for neglecting the effects of air speed, Ehrlemark and Sällvik (1996) used a similar index [the thermal load index (TLI); Ehrlemark and Sällvik (1996)] for normalizing the environmental conditions and comparison with experimental data and thus failed to address the shortcoming they had identified.

Acknowledging the practical difficulties arising from the complexity of McArthur’s model, Turnpenny et al. (2000a) presented a simplified version of the model, dubbed “parsimonious,” which was applied to various livestock [cattle, sheep, pigs, chickens (Turnpenny et al. 2000b)]. Following the same general approach, McGovern and Bruce (2000) developed a transient model which also included thermoregulatory mechanisms, namely reducing the thermal resistance of body tissue (vasodilation), sweating to increase latent heat loss and panting to increase respiratory heat loss. An accompanying algorithm for time-step simulations was also presented (McGovern and Bruce 2000). Similarly, Thompson et al. (2014) developed a comparable transient model, including relatively detailed climate submodels for calculating the ambient temperature, wind speed, and solar radiation, to be used when hourly weather data is not available. Li et al. (2021) added a submodel for conduction between a lying animal and the ground to the model by Thompson et al. (2014), further increasing its complexity. The model has so far only been used to perform a sensitivity analysis (Li et al. 2021). As mentioned above, none of these models has been systematically applied to identify conditions of potential heat stress, especially in terms of common meteorological parameters.

A general shortcoming of the mentioned heat-balance models is lack of validation against experimental data. Although most submodels used to describe individual thermal or physiological phenomena are well established, none of the whole models is thoroughly validated by comparison with reliable measurements. For instance, Ehrlemark and Sällvik (1996) compared the predictions of their model with experimental data pooled from several sources. Notably, the model predictions were in poor agreement with the experimental data at higher ambient temperatures, namely conditions of potential heat stress. Moreover, as mentioned above, the results were only presented in terms of a thermal load index (TLI) that obscures important boundary conditions such as the air speed. Recently, more attention has been paid to addressing the validation gap. Li et al. (2021), for example, compared their modeling results with experimental data from the literature, although with unsatisfactory results. The model overpredicts the core body temperature by up to 3°C which, given the physiological thresholds for heat stress, seems too large. On the other hand, the respiration rate, estimated based on linear regression with the body temperature, was underpredicted almost systematically, by up to 40% (60 br/min discrepancy at 140 br/min).

Turnpenny et al. (2000b) observed that the limited data available on the partition of heat loss, heat generation, and the thermophysical characteristics of the livestock hinders further development and refinement of heat-balance models. Two decades later, that limitation remains the case. Despite calorimetric methods, specifically measurements in respiration chambers, being well established and widely used for several decades, detailed measurements of heat transfer and thermoregulation in dairy cattle is scarce. In many widely used sources, the thermoneutral metabolic heat generation rate, a key boundary condition in heat-balance models, is calculated and reported as the residual value of energy-portioning calculations focusing on nutrition and productivity, e.g. Coppock (1985); van Knegsel et al. (2007); Talmón et al. (2020). On the other hand, thermodynamic measurements of thermoregulation which deal with the details of heat partition were mostly conducted decades ago, on animals with much lower yield than the present-day high-yielding cow, with presumably lower metabolic heat generation and possibly different thermophysiological characteristics. The seminal study by Worstell and Brody (1953), for example, was conducted on Holstein cows whose mean milk yield was less than 20 kg/day. The similarly widely referenced study by Purwanto et al. (1990) was conducted on cows among which the “high-yielding” group had a mean milk yield of about 30 kg/day. More recent measurements of respiratory and cutaneous heat losses by Maia et al. (2005a, b) were performed on pasture-fed Holsten cows with even lower yield, 15 kg/day on average. The existing models, including those referenced above, all rely on such historical data, while the modern Holstein-Friesian dairy cow can have yields averaging around 40 kg/day (Pinto et al. 2020) and exceeding 50 kg/day during peak lactation.

Perhaps driven by an increasing awareness of the endemic validation gap, there has recently been a slow resurgence of attention to the measurement of heat transfer and thermoregulatory responses in cattle. The recent thermographic study of the skin temperature by Yan et al. (2021) is a promising step forward, although the presentation of the results follows the THI orthodoxy. Another important development is the work of Zhou et al. (2021) where the physiological and productive responses of dairy cattle to various combinations of ambient temperature, humidity, and air speed were measured in a respiration chamber. Nevertheless, Zhou et al. (2021) do no present the partitioning of heat dissipation into different modes of heat transfer.

Another general disadvantage of the mechanistic heat-balance models is their formal complexity. Mechanistic models such as the heat-balance models discussed here have been criticized as unsuitable for use in precision farming due to complexity and presence of many parameters that often need to be re-evaluated or adjusted for each application (Wathes et al. 2008). Nevertheless, formal complexity and the resulting computational intensity are not necessarily as big a barrier to wide application as they were 20 or even 10 years ago. As pointed out by Stiehl and Marciniak-Czochra (2021), the present day’s computational power allows the investigation of rather complex issues based on mechanistic models, affording a deep quantitative understanding of a wide range of topics. Aside from the simplified general model by Turnpenny et al. (2000a, b), no effort has been made to address the implementation gap for heat-balance models of cattle, particularly to facilitate implementation in predictive-model control of the barn climate. Furthermore, as mentioned above, no attempt has been made at systematic application of such models to identify conditions of potential heat stress and develop meteorological indices.

## Parallels with human biometeorology

### Historical development

The ongoing research on heat stress in cattle exhibits several parallels with human biometeorology, specifically the development of human thermal comfort indices. Given the longer history and relatively greater success of the latter, there are lessons to be learned for a more effective pursuit of the former. After all, the most widely used index, THI, was imported from human meteorology, virtually shaping the trajectory of research on heat stress in cattle for more than half a century. The striking parallels between research on thermal comfort and heat stress in humans and cattle are therefore no coincidence. In both cases, researchers started with a focus on the ambient temperature, then developing statistical constructs that incorporated humidity, air speed, and finally solar radiation as wider ranges of climates and production facilities were considered. The parallels are demonstrated, for instance, by a recent study (Kovács et al. 2018) where the apparent temperature (for humans) was found to be a better predictor of heat stress in dairy calves than several variants of THI.

In 1962, about the time THI was imported into animal science, Macpherson (1962) published a comprehensive review of the metrics devised to assess thermal comfort in humans. Interestingly, Macpherson’s 1962 review enumerates 19 indices for human thermal comfort; Ji et al.’s 2020a review lists 20 heat stress indices for dairy cattle. As documented in by Macpherson (1962), the pioneering work on human thermal comfort was by large inspired and motivated by the proliferating industrial plant; industrial and mechanized animal husbandry has likewise enhanced the need for effective prediction and remediation of heat stress.

Judging from the large number of heat stress indices developed during the half century prior, Macpherson (1962) concluded that there was simultaneously a great need for quantifying and categorizing conditions of thermal comfort, and little success through the means thitherto devised. Most notably, Macpherson (1962) concluded:

“[A]ssessment of the thermal environment is not primarily a matter of the selection of some thermal index in which to express the results. Expressing the results in the form of an index may be a convenience, but the assessment of the environment is essentially the measurement of all the factors concerned… Indices of thermal stress do not provide a substitute for a sound knowledge of the mechanisms of heat exchange and of the physiological adjustments to the thermal environment.”

The current state of the art in dairy science suggests a similar conclusion.

### Apparent temperature revisited

Inspired by the conclusion that a suitable heat index would be based on the heat balance of the human body (Macpherson 1962), Steadman (1979a, b, 1984) presented a meticulous analysis of heat transfer from the human body to derive an equivalent temperature. Dubbed the “apparent” temperature, this equivalent temperature is defined as the dry-bulb temperature at standardized humidity, wind speed, and radiation, which would require the same thermal resistance for a walking adult to feel thermal comfort under a given set of meteorological conditions. The procedure used to derive the apparent temperature, *T*_ap_, is as follows. The heat-balance equation is first solved iteratively for various sets of environmental conditions to obtain the thermal resistance of clothing, *R*_f_, required for thermal balance. This first solution is based on the idea that, in equilibrium, postulated by Steadman (1979a) as a condition of thermal comfort, the total heat dissipation rate should equal the heat generation rate, less the net effect of the incoming solar, albedo and terrestrial radiation and the outgoing sky radiation (Steadman 1979a, b). Moreover, the core body temperature, *T*_b_, is assumed constant at 37°C as a condition of comfort. After obtaining *R*_f_, *T*_ap_ is determined by solving the heat-balance equation for *T*, this time with *R*_f_ known from the first solution, and with the other meteorological parameters (solar radiation, air speed, and humidity) set as “standard,” i.e. reference, values.

Perhaps most relevant to the topic of heat stress in cattle and the slew of statistical indices is the demonstration (Steadman 1979b) that for any heat index, *T*_x_, constructed as a linear combination of the dry-bulb temperature (*T*_db_), wet-bulb temperature (*T*_wb_), and globe temperature (*T*_gt_), i.e.^2^

$${T}_x={c}_1{T}_{\mathrm{db}}+{c}_2{T}_{\mathrm{wb}}+{c}_3{T}_{\mathrm{gt}}+{c}_4$$

the coefficients *c*_1_, *c*_2_, and *c*_3_ are *not constant*, but highly dependent on the ambient temperature and considerably dependent on activity level (heat generation), humidity, and wind speed (Steadman 1979b). This key observation explains the failure of indices constructed using regression analysis of environmental and physiological, behavioral, or productive parameters. The constant coefficients typically obtained from such analyses confine the proposed index to the environmental conditions and physiological characteristics covered in the original study. See for instance the recent work of Lees et al. (2022) which concluded that DHLI, which was developed to include the effects of solar radiation, is a better predictor of heat stress than THI (no radiation term), but only for unshaded cows. Ehrlemark and Sällvik (1996) duly observed that the validity of the statistical models is limited by the range of conditions covered by the underlying experimental data. Compare the general analytical expressions for *c*_1_, *c*_2_, and *c*_3_ derived by Steadman (1979b) and the definitions of common heat stress indices for cattle, summarized by Ji et al. (2020a).

Recognizing the need for simplified calculation of *T*_ap_, Steadman (1984) also presented linear equations based on multiple-regression analysis of computed values of *T*_ap_. There have subsequently been other efforts to develop simpler approximations that entail fewer independent variables, e.g. only *T*_db_ and RH (Rothfusz 1990). It is important to note, however, that such regression-based equations are ultimately based on thermodynamic models, i.e. the heat balance of the animal, rather than on purely statistical correlations between observations of arbitrary predictors and indicators. Moreover, in constructing *T*_ap_, several conditions were imposed such that the result would have physical meaning and significance. For instance, it was observed that an equivalent temperature used to express comfort “must have the familiar properties of temperature” (Steadman 1984). As mentioned above, some of the cattle heat stress indices do not satisfy such criteria.

In general, Steadman’s model for humans is simpler than heat-balance models for cattle due to several underlying simplifying assumptions that are not applicable to cattle. In Steadman’s model, the basic link between the actual environmental conditions and the standard conditions at which *T*_ap_ is evaluated is the thermal resistance of clothing (*R*_f_). The fundamental assumption of the model is that, outdoors, humans seek (achieve) thermal comfort by adjusting clothing, more precisely the heat and moisture transfer resistance of clothing. This fundamental feature cannot be extended to animals, e.g. dairy cattle, since the haircoat is relatively constant, despite seasonal variations that decrease the heat and moisture transfer resistance of the coat in summer and vice versa in winter (Façanha et al. 2010). Furthermore, the assumptions and submodels used by Steadman (1979a, b) for evaluating the various heat and vapor transfer resistances do not apply to cattle. Lastly, it is important to note that the apparent temperature scale was developed based on a constant activity level, representing an adult Caucasian walking at 1.4 m/s (Steadman 1984). It must also be noted that research on human thermal comfort and perception goes on, with heat-balance models continuing to provide the framework for many influential developments. See, for instance, the review by Rupp et al. (2015).

## Thermodynamic assessment of heat stress in dairy cattle

In this section, a model from the literature is applied following the general approach used to develop *T*_ap_ to demonstrate the utility of heat-balance models in predicting heat stress, to derive meteorological indices, and to develop a general framework for systematic application of such models.

### Assumptions and procedure

The general livestock heat-balance model developed by Turnpenny et al. (2000a, b) was used where the total heat dissipation from the animal, *G*_e_, is estimated and compared with the thermoneutral metabolic heat generation rate, *M*, both expressed in terms of heat flux, per unit skin surface area. In general, *G*_e_ is comprised of sensible and latent heat loss from the skin and through respiration. With no solar radiation, thermal balance (equilibrium) is maintained when *G*_e_=*M.*

The thermoregulatory responses are iteratively adjusted to find conditions where *G*_e_=*M*, following the “principle of least metabolic cost” (Mount 1974; Turnpenny et al. 2000a), namely that an animal will use vasomotor control before increasing evaporative heat loss which involves water loss and/or an increase in metabolic rate. This means that, for given boundary conditions, thermoregulation is simulated by:

1. Decreasing the tissue resistance to heat transfer (*r*_s_), simulating vasodilation, until thermal balance is achieved (*G*_e_=*M*). There is a physiological lower limit to this resistance.
2. If the minimum tissue resistance is not sufficient for thermal balance, i.e. *G*_e_<*M*, the cutaneous latent heat loss (sweating) is increased until thermal balance is achieved. There is an upper limit to this heat loss mechanism, dictated by either physiology or the environment.
3. In the present model, the respiration rate was independently calculated, based on the ambient temperature and humidity and using empirical correlations.
4. If the maximum sweating rate is not sufficient to maintain balance, i.e. *G*_e_<*M*, heat will accumulate in the body and the core body temperature will increase.

Note that, as pointed out by McArthur (1987), there is evidence that sweating starts before tissue resistance has been minimized, i.e. thermoregulation through sweating and vasodilation may occur simultaneously and not necessarily in succession. Nevertheless, the step-by-step model of Turnpenny et al. (2000a) provides a reasonable first approximation.

Further note that:

- Metabolic heat generation was assumed constant at the thermoneutral rate. In reality, the metabolic rate declines with prolonged exposure to heat, thereby increasing the animal’s tolerance to heat. This adjustment is achieved by reduced food intake and thyroid gland activity. See the paper by McArthur (1987) and the references therein cited for details.
- Similarly, the core body temperature, *T*_b_, was assumed constant at the thermoneutral level (39°C).
- As will be shown, the skin temperature is a *dependent* variable, i.e. not directly adjusted as a thermoregulatory response.
- Only the *onset* of bodily heat accumulation is sought. Therefore, the increase in *T*_b_ and reduction of *M* as secondary coping mechanisms were not modeled.

Some of the sub-models used here are slightly different from those used by Turnpenny et al. (2000a, b). Details of the model implementation including boundary conditions, submodels, and validation against experimental data are presented in the Appendix.

### Sample modeling results

**Fig. 1:**
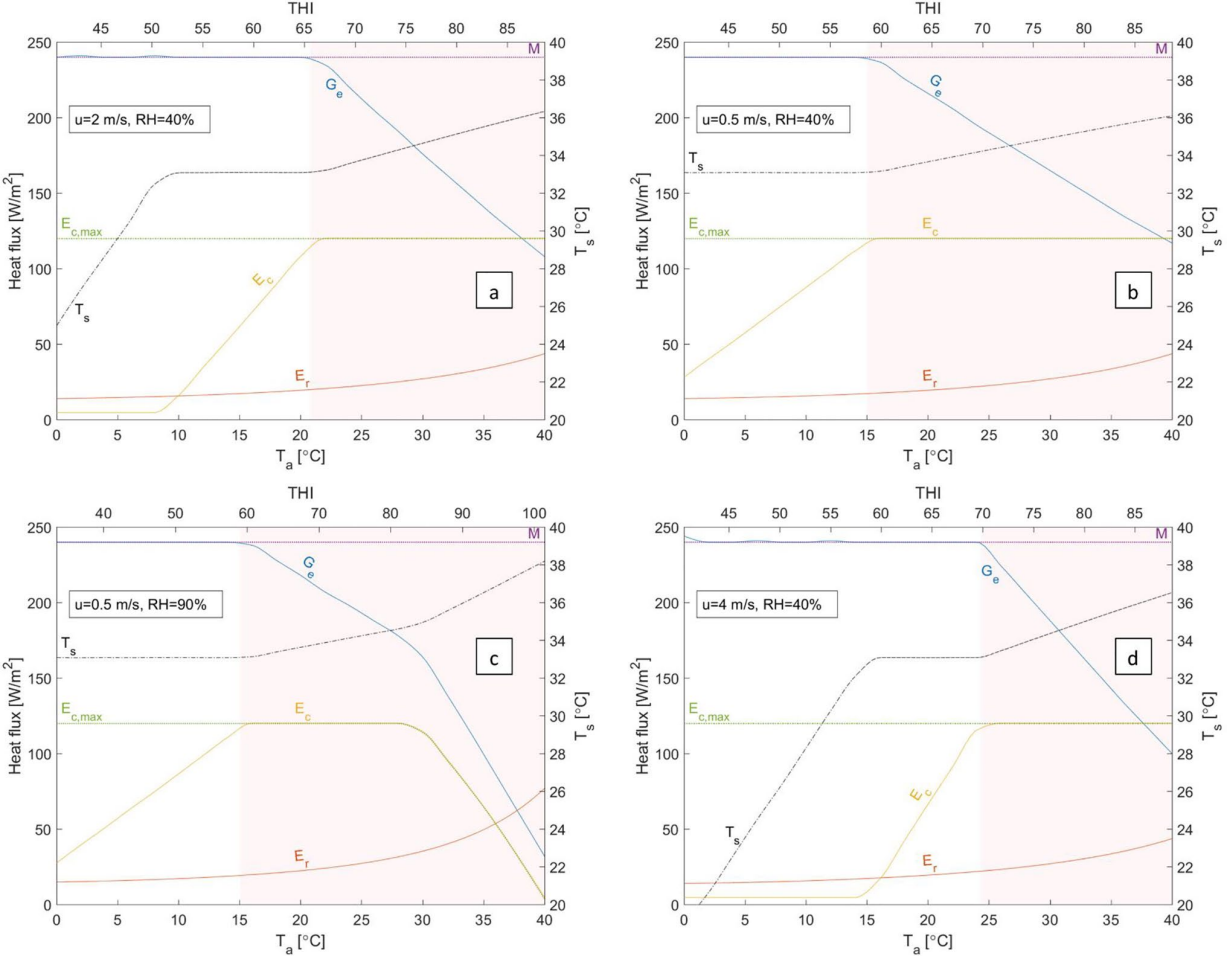
Equilibrium heat fluxes and skin temperature of a Holstein dairy cow as a function of the ambient temperature and for various air speed and relative humidities and no solar radiation, estimated based on the heat-balance model of Turnpenny et al. (2000a). *M*, metabolic heat generation; *G*_e_, total heat dissipiation; *E*_c_, cutaneous latent heat loss; *E*_r_, repsiratory heat loss; *T*_s_, skin temperature. Shaded area denotes *G*_e_<*M* where heat accumulates in the body and *T*_b_=const and *M*=const assumptions may no longer be valid

Figure 1 shows sample results obtained from running the heat-balance model at sample constant air speed and relative humidity, and for various values of the ambient temperature, *T*_a_. The main output to observe is *G*_e_, specifically its magnitude relative to *M*. As mentioned above, bodily heat accumulation starts when *G*_e_<*M*. Latent heat flux from the skin is denoted by *E*_c_ while the respiratory heat flux (dominantly latent) is denoted by *E*_r_. In the present model, *E*_r_ is a function of the *T*_a_ and RH only, i.e. adjusted independently of vasodilation and sweating. See the Appendix for details. The third component of *G*_e_, not shown in Fig. 1, is the sensible heat flux from skin (convection and long-wave radiation).

Figure 1a suggests that, for *u*=2 m/s and RH*=*40%, the thermoregulatory responses are sufficient to maintain the heat balance up to just above 20°C. In other words, increasing *T*_a_ to ~20°C, vasodilation and sweating are able to reduce the overall heat transfer resistance between the body core and the ambient in order to compensate for the reduction in *T*_b_-*T*_a_. The thermoregulatory responses in this region (*T*_a_ ≲ 20.5°C) can be divided to two phases:

1. For *T*_a_ ≲ 8°C, vasodilation is sufficient for maintaining *G*_e_=*M*, as seen from the increase in the skin temperature, *T*_s_, plotted against the vertical axis on the right. (Because *M* and *T*_b_ are constant, reducing *r*_s_ increases *T*_s_; see Eq. (A4) in the Appendix.)
2. After *r*_s_ reaches its physiological minimum, i.e. vasodilation exhausted, sweating is enhanced to dissipate more latent heat from the skin; this is reflected by the monotonic increase of *E*_c_ for 8°C ≲ *T*_a_ ≲ 20.5°C. The linear increase in *E*_c_ counteracts the linear decrease of the sensible heat flux, caused by the decrease in the heat transfer potential (*T*_b_-*T*_a_). The slope of both lines is the convection heat transfer coefficient, proportional to a power of the air speed. See Eq. (A6) in the Appendix.

For *u*=2 m/s and RH*=*40%, the onset of heat accumulation at *T*_a_≈20.5°C is dictated by *E*_c_ reaching its limit, in this case *E*_c,max_=120 W/m^2^. As discussed in the Appendix, this limit can be physiological or environmental.

In reality, once *r*_s_=*r*_s,min_ and *E*_c_=*E*_c,max_, *T*_b_ increases, followed by a decrease in *M* due to reduced feed intake and metabolic activity^3^. The results shown in Fig. 1a are therefore only qualitatively valid for *T*_a_ ≳ 20.5°C, nonetheless instructive. The shaded area denotes *G*_e_<*M*.

The effect of air speed can be seen from comparison of Fig. 1a, b, and d. Higher air speed, corresponding to a higher convective heat loss from the skin, shifts the onset of heat accumulation to a higher *T*_a_. In other words, vasodilation (reflected by the rise in *T*_s_) and sweating (reflected by the rise in *E*_c_) are triggered and therefore exhausted at higher *T*_a_, meaning *G*_e_=*M* can be maintained for higher values of *T*_a_.

Most notably, Fig. 1 suggests that humidity has little effect on the onset of heat accumulation. Compare Fig. 1b and c, representing moderate (RH*=*40%) and extremely high (RH*=*90%) humidity, respectively. Note how the various heat fluxes are virtually identical up to *T*_a_≈27°C, well beyond the onset of heat accumulation (*T*_a_≈15°C in both cases). Even at *u*=0.5 m/s, corresponding to “clam” air (WMO 1970), *E*_c_ reaching its physiological limit is the determining factor. The adverse effect of excessive humidity (RH*=*90%; Fig. 1c) on heat dissipation becomes apparent only at *T*_a_ ≈27°C, some 12°C above the onset of heat accumulation, where *E*_c_ is suppressed below the physiological limit, leading to a drastic drop in *G*_e_. This is in agreement with the conclusion by Turnpenny et al. (2000b) that evaporative heat loss from cattle is restricted by ambient vapor pressure only at high ambient temperatures (*T*_a_>30°C), while at lower ambient temperatures, sweating is limited by water supply rather than environmental conditions. Similarly, the effect of humidity on *E*_r_ is only significant for *T*_a_ ≳ 30°C, as seen from Fig. 1a and c.

## From heat fluxes to temperatures

As shown above, the main outcome of heat-balance models is heat dissipation fluxes which can then be compared to fluxes from the heat sources (metabolic heat generation and solar irradiation) to assess thermal balance. Nevertheless, heat fluxes are not as tangible as meteorological parameters (ambient temperature, humidity, air speed). Moreover, as discussed in Heat stress: Indicators and predictors, effective implementation for heat stress prevention/alleviation depends on indices and thresholds in terms of the readily available meteorological parameters. This section presents three proposals for translating the heat-flux results into simplified indices for the state of thermoregulation and the onset of heat accumulation.

### Onset of heat accumulation: the critical temperature

In order to express the heat-balance results in terms of the more familiar meteorological parameters, a critical temperature, *T*_cr_, may be defined as the ambient temperature corresponding to the onset of heat accumulation^4^ for given *u* and RH. Here, *T*_cr_ was defined as *T*_a_ for which *G*_e_ falls to 99% of *M*, an arbitrary threshold.

**Fig. 2:**
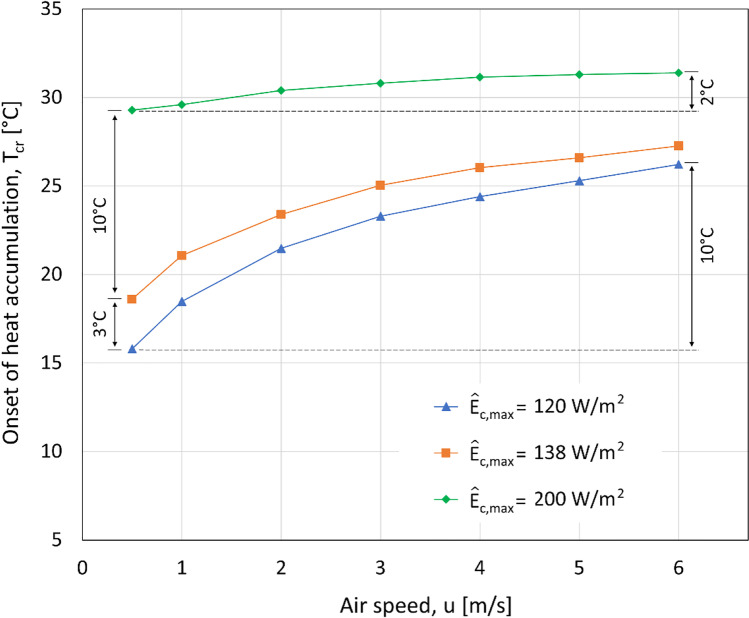
Critical tempearture as function of air speed for various representative phsyiological limits on sweating (*M* = 240 W/m^2^, no solar radiation)

In Fig. 2, *T*_cr_ is plotted as a function of *u* for RH*=*40% and three representative values of the physiological limit on *E*_c_, denoted by ${\hat{E}}_{\mathrm{c},\max }$:

1. ${\hat{E}}_{\mathrm{c},\max }=120\ \mathrm{W}/{\mathrm{m}}^2$ corresponding to historical data for Holstein cows, used by Turnpenny et al. (2000b), also the default value in the present model.
2. ${\hat{E}}_{\mathrm{c},\max }=138\ \mathrm{W}/{\mathrm{m}}^2$ corresponding to the average of the measurements by Gebremedhin et al. (2010) for Holstein cows.
3. ${\hat{E}}_{\mathrm{c},\max }=200\ \mathrm{W}/{\mathrm{m}}^2$ corresponding to the upper end of the measurements by Gebremedhin et al. (2010). This is an extremely high heat flux, unlikely to be sustained over the entire skin area, especially at *M* = 240 W/m^2^. This value was nonetheless included for demonstration and comparison purposes.

Recall that, as discussed in the “Sample modeling results” section, humidity hardly has any effect on the *onset* of heat accumulation. The significant effect of air speed (*u*), on the other hand, can be seen in Fig. 2, especially for ${\hat{E}}_{\mathrm{c},\max }=120\ \mathrm{W}/{\mathrm{m}}^2$ and ${\hat{E}}_{\mathrm{c},\max }=138\ \mathrm{W}/{\mathrm{m}}^2$: increasing the air speed from 0.5 to 6 m/s, shifts *T*_cr_ by as much as 10°C. For ${\hat{E}}_{\mathrm{c},\max }=200\ \mathrm{W}/{\mathrm{m}}^2$, the excessive sweating capacity can compensate reduced convective heat loss at lower air speeds to a large extent, diminishing the difference between *T*_cr_ at *u*=0.5 m/s and *u*=6 m/s. Noteworthy is that, according to Fig. 2, heat accumulation may start well below the established values for the upper critical temperature (UCT), e.g. 25°C according to Berman et al. (1985), especially for *u* < 4 m/s. The *T*_cr_ results are in agreement with UCT=25°C only for animals with extremely high sweating capacity (${\hat{E}}_{\mathrm{c},\max }=200\ \mathrm{W}/{\mathrm{m}}^2$) or at high air speeds (*u* > 4 m/s).

### Apparent temperature for cattle

The next step in consolidating the results would be to integrate *T* and *u* into a single parameter. Following the general procedure used by Steadman (1979a, b, 1984) to develop *T*_ap_, an “apparent temperature” for cattle, ${\overset{\sim}{T}}_{\mathrm{ap}}$, may be defined for any combination of *T*_a_ and *u* as the ambient temperature which would require the same *E*_c_ for thermal equilibrium at a reference air speed.^5^ The main difference with *T*_ap_ (Steadman 1984) is that, instead of the required clothing insulation, *E*_c_ is the link between the actual and apparent temperatures. Since air is rarely still in naturally ventilated dairy barns, *u*_0_=1.4 m/s was chosen as the reference air speed, corresponding to the upper end of the “light air” range in the Beaufort scale (WMO 1970) and in accordance with the original *T*_ap_ (Steadman 1984; see the “Apparent temperature revisited” section).

**Fig. 3:**
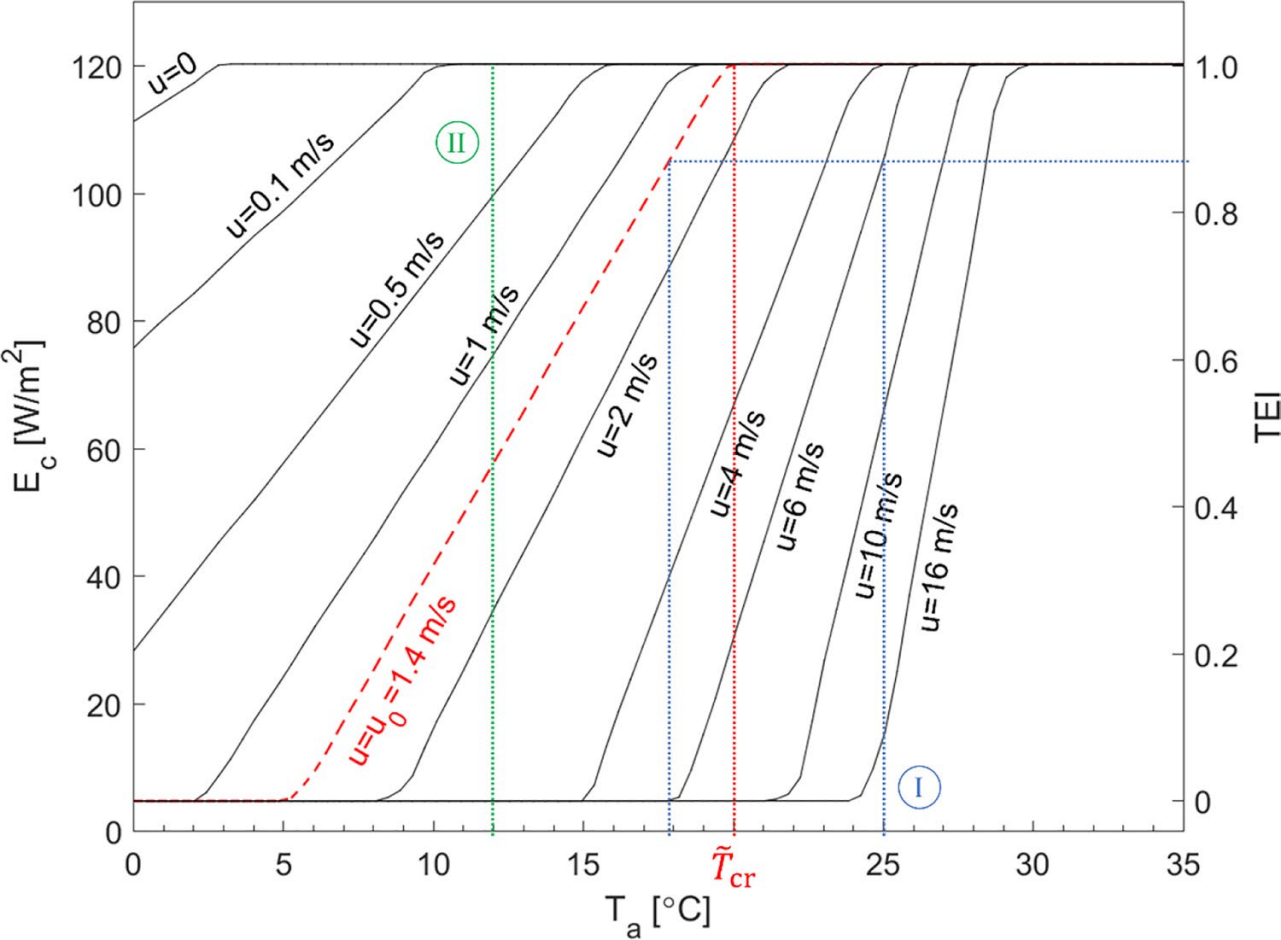
Equilibrium cutaneous latent heat flux (*E*_c_) and thermoregulatory exhaustion index (TE) as functions of ambient temperature and at various air speeds; graphical evaluation of apparent temperature for cattle (${\overset{\sim }{T}}_{\mathrm{ap}}$) [*M* = 240 W/m^2^, ${\hat{E}}_{\mathrm{c},\max }$ = 120 W/m^2^, no solar radiation]

The procedure for calculating ${\overset{\sim }{T}}_{\mathrm{ap}}$ is demonstrated in Fig. 3 where the equilibrium *E*_c_ is plotted as a function of *T*_a_ and for various values of *u*. For any given combination of *T*_a_ and *u*, there is a unique equilibrium *E*_c_ so long as *T*_a_<*T*_cr_. To find ${\overset{\sim }{T}}_{\mathrm{ap}}$, the corresponding line of constant *E*_c_ is intersected with the *u*_0_ curve (dashed curve in Fig. 3); ${\overset{\sim }{T}}_{\mathrm{ap}}$ is the abscissa of the intersection.

Even in the absence of strong winds, there is air movement around caused by free convection and the movements of the animals. The *u*=0 curve was therefore included in Fig. 3 only as a reference for comparison. On the other hand, *u*=16 m/s represents “near gale” high winds (WMO 1970), also included for comparison. In most applications, the air speed is between 0.5 and 6 m/s.

### Thermoregulatory exhaustion index

From Fig. 2, it is known that *T*_cr_ =20°C for *u*=1.4 m/s and *E*_c,max_ = 120 W/m^2^. Transformed into ${\overset{\sim }{T}}_{\mathrm{ap}}$, any combination of *T*_a_ and *u* can simply be compared against this threshold, ${\overset{\sim }{T}}_{\mathrm{cr}}={T}_{\mathrm{cr}}\left({u}_0\right)={20}^{{}^{\circ}}\mathrm{C}$, to determine whether thermonetural heat balance can be maintained as well as how far the conditions from the onset of heat accumulation are. A second integrated parameter can be defined to characterize the extent to which the thermoregulatory mechanisms (particularly sweating) have been “exhausted” and as a measure of how far the conditions from the upper limit of the thermoneutral zone are. Here, the thermoregulatory exhaustion index (TEI) is defined as:

$$\mathrm{TEI}=\frac{E_{\mathrm{c}}-{E}_{\mathrm{c},\min }}{E_{\mathrm{c},\max }-{E}_{\mathrm{c},\min }}$$

where *E*_c,min_ and *E*_c,max_ denote the minimum and maximum of *E*_c_, respectively.^6^

One advantage of TEI over *T*_cr_ is that it entails two universal limits, TEI=0 and TEI=1.0 correspoding to *E*_c_= *E*_c,min_ and *E*_c_= *E*_c,max_ respectively. In other words, TEI consolildates the effect of the most important enviornmental (*T*_a_, *u*) and phsyiological parameters (*E*_c,max_). TEI=1.0 signifies the exhaustion of the “primiary” thermoregulatory respnse mechanisms and the onset of heat accumulation.

In Fig. 3, TEI is plotted against the vertical axis on the right. Two examples are also shown:

$$\begin{array}{c}{T}_{\mathrm{a}}={25}^{\circ}\mathrm{C},u=6\ \mathrm{m}/\mathrm{s}\Rightarrow {E}_{\mathrm{c}}\approx 106\ \mathrm{W}/{\mathrm{m}}^2\\ \Rightarrow {\overset{\sim }{T}}_{\mathrm{a}\mathrm{p},\mathrm{I}}\approx {18}^{{}^{\circ}}\mathrm{C}<{\overset{\sim }{T}}_{\mathrm{c}\mathrm{r}},{\mathrm{TEI}}_{\mathrm{I}}=0.88\end{array}$$

$$\begin{array}{c}{T}_{\mathrm{a}}={12}^{\circ}\mathrm{C},u=0.1\ \mathrm{m}/\mathrm{s}\Rightarrow {E}_{\mathrm{c}}=120\ \mathrm{W}/{\mathrm{m}}^2\\ \Rightarrow {\overset{\sim }{T}}_{\mathrm{a}\mathrm{p},\mathrm{II}}>{\overset{\sim }{T}}_{\mathrm{c}\mathrm{r}},{\mathrm{TEI}}_{\mathrm{II}}=1.0\end{array}$$

In Example I, ${\overset{\sim }{T}}_{\mathrm{ap},\mathrm{I}}<{\overset{\sim }{T}}_{\mathrm{cr}}$, meaning *G*_e_=*M* can be sustained and therefore thermoneutrality maintained, reflected by TEI_I_<1.0. On the other hand, in Example II, *E*_c_ = *E*_c,max_, meaning the thermoregulatory reponses are exausted (TEI_II_ = 1.0) beyond thermoneutrality and heat accumulation is underway.

Note that the results shown in Fig. 3 apply to the representative values of the physiological parameters, particularly *M* and ${\hat{E}}_{\mathrm{c},\max }$, used in the present model. Similar graphs can be generated for animals with different characteristics or other weather conditions of interest, including with solar radiation.

## Conclusion

Indices constructed through regression of meteorological parameters and animal responses have dominated research on heat stress in cattle for more than six decades. Nevertheless, there is increasing skepticism about the effectiveness and adequacy of such indices. As suggested by the parallels with human biometeorology drawn in this paper, forging new paths forward to meet the needs of modern livestock management in the times of climate change requires a renewed attention to physics-based heat-balance models. Although several heat-balance models have been developed, no attempt has been made at systematic application of the models to predict conditions of potential heat stress.

In that context, the present work revisited classical work in human biometeorology to develop a framework for identifying heat stress based on thermodynamic models of thermoregulation and heat dissipation. A model from the literature was used to assess the heat balance of a typical Holstein dairy cow under various combinations of ambient temperature, humidity, and air speed. It was shown that the onset of heat accumulation strongly depends on temperature and air speed, but hardly on humidity. While many studies have paid little attention to the effect of air speed on heat stress, especially in the vicinity of the animal, the results of the present study underline the importance of systematic collection and reporting of air speed data.

As evidenced by the dominance and resurgence of THI and its many variants, an easy-to-use index presented in tables or simple graphs is extremely useful. Simplicity and ease-of-use compel practitioners and researchers to be surprisingly forgiving of fundamental and methodological shortcomings. Therefore, the present work introduced new parameters to translate the modeling results (heat fluxes) into meteorological indices. The critical temperature denotes the onset of heat accumulation at given air speed. The apparent temperature for cattle maps the ambient temperature at any given air speed onto a thermophysiologically equivalent temperature at a reference air speed. Finally, the thermoregulatory exhaustion index (TEI) is a measure of the extent to which the thermoregulatory responses have been mobilized and how far the conditions from the upper limit of the thermoneutral zone are.

The general framework developed in this paper can serve as a roadmap for future work. To further establish the thermodynamic approach, the endemic validation gap must be first addressed through detailed measurements of the thermophysiological characteristics of the modern high-yielding cow, most importantly the thermoneutral metabolic heat generation rate and core boy temperature, and the maximum sweating rate. Furthermore, concrete definitions for what constitutes heat stress must be developed in physiological or productive terms. More precisely, thresholds for critical heat strain, e.g. in terms of increase in the core body temperature or decrease in milk yield, must be established. Finally, metabolism and productivity must be integrated in the thermodynamic heat dissipation models in order to increase the utility and accuracy of the models for analysis at animal-individual level.

## Figures and Tables

**Figure A1:**
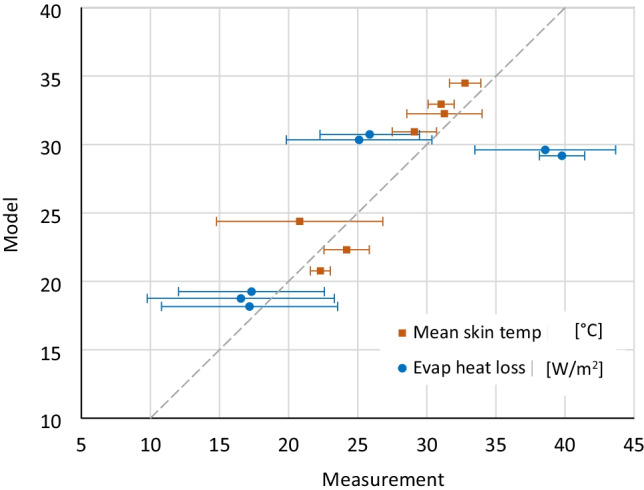
Comparison of the presnet model’s predictions for the mean skin temperature and total evapoartive (latent) heat loss with the respiration chamber measurements of Blaxter and Wainman (1964) for steers (see Table A1)

## Appendix: Details of the heat balance model

### General approach and formulation

The heat-balance model by Turnpenny et al. (2000a,b) was implemented where the total heat dissipation from the animal, *G*_e_, is estimated, and compared with the thermoneutral metabolic heat generation, *M*. When the skin surface is in thermal equilibrium, the total heat flux dissipated to the environment equals the heat flux through the body tissue, *G*_s_, as well as the heat flux through the haircoat, *G*_c_;

$${G}_{\mathrm{e}}={G}_{\mathrm{s}}={G}_{\mathrm{c}}$$

For given environmental conditions (ambient temperature, humidity, air speed, solar radiation), heat-balance equations are solved iteratively to find the equilibrium skin and coat temperatures. The thermoregulatory responses are incrementally increased during iterations until converged. See the papers by Turnpenny et al. (2000a, b) for more details and general solution algorithm.

Cattle dissipate bodily heat mainly through respiration and from skin. The total heat dissipation rate on a flux (per unit area) basis can be written as shown in Eq. (A1) (Turnpenny et al. 2000a).

A1 $${G}_{\mathrm{e}}=\frac{\rho {c}_{\mathrm{p}}}{r_{\mathrm{H}}}\left({T}_{\mathrm{c}}-{T}_{\mathrm{a}}\right)+\frac{\rho {c}_{\mathrm{p}}}{r_{\mathrm{R}}}\left({T}_{\mathrm{c}}-{T}_{\mathrm{r}}\right)-{S}_{\mathrm{a}\mathrm{bs}}+{E}_{\mathrm{r}}+{E}_{\mathrm{c}}$$

where *G*_e_ is the heat flux per unit skin surface area; the first term on the right-hand side represents convective heat transfer from the haircoat to the ambient air; the second term represents the net long-wave radiant exchange at the external surface of the haircoat; *S*_abs_ is the absorbed solar radiation; *E*_r_ is the dominantly latent heat flux through respiration; and *E*_c_ is the latent heat flux from the skin, both normalized by the skin surface are, *A*_s_.

Following McArthur (1987), the formulation of Monteith and Unsworth (1990) was used for the sensible components of heat loss:

A2 $$G=\frac{\rho {c}_{\mathrm{p}}\Delta T}{r}$$

where Δ*T* is the driving temperature difference, *r* [s/m] is the resistance to heat transfer, and *ρ*c_p_ is constant, here taken as 1220 J/(m^3^K), corresponding to 20°C and 1 atm.

### Animal data

The animal size characteristics were chosen to represent a typical mature Holstein cow: *m*=670 kg, *d*_t_=0.5 m. A haircoat length of *l* =9 mm was chosen to represent the summer conditions (Façanha et al. 2010).

The skin surface area, *A*_s_ [m^2^], was calculated using the regression proposed by Webster (1974):

A3 $${A}_{\mathrm{s}}=0.09\ {m}^{0.67}$$

The result is in agreement with the measurements of Le Cozler et al. (2019) though not with the conclusion by Berman (2003).

The model by Turnpenny et al. (2000b) was originally developed as a generic model for livestock, including sheep, pigs, cattle, and poultry. Consequently, the model entails parameters to account for the difference between the skin surface area and the haircoat surface area as well as the haircoat’s effect on the curvature of the exposed surface. Given that *l* is small for cattle, those two effects were ignored in the present work. Furthermore, the entire skin area was assumed to be exposed, representing a cow in standing position and thermally isolated from other animals. The latter assumption is supported by the conclusion by Wiersma and Nelson (1967) that convective heat loss is not affected by the presence of animals farther than two inches (approximately five centimeters).

The metabolic heat flux, *M* [W/m^2^], was calculated based on the regression proposed by van Knegsel et al. (2007) for daily heat production in lactating cows, 1110 *m*^0.75^ [kJ], resulting in an average metabolic heat flux of *M*=240 W/m^2^. More recent measurements by Talmón et al. (2020) yield comparable results for pasture-fed Holstein cows.

Following Turnpenny et al. (2000b), the core body temperature was assumed constant at *T*_b_=39°C.

### Ambient conditions

Ambient conditions were defined in terms of pressure, *p* [kPa], temperature, *T*_a_ [°C], mean radiant temperature, *T*_r_ [°C], relative humidity, RH [%], wind speed, *u* [m/s], and assuming no solar irradiation. For simplicity it was assumed *T*_r_=*T*_a_, though it is likely that *T*_r_ >*T*_a_ during hot sunny days.

### Heat transfer through body tissue

The rate of heat transfer through body tissue is given by:

A4 $${G}_{\mathrm{s}}=\frac{\rho {c}_{\mathrm{p}}}{r_{\mathrm{s}}}\left({T}_{\mathrm{b}}-{T}_{\mathrm{s}}\right)$$

where *r*_s_ [s/m] is the resistance of the body tissue to heat transfer, *T*_b_ is the core body temperature, and *T*_s_ is the mean temperature of the skin surface.

The vasomotor response is simulated by adjusting *r*_s_, starting at 100 s/m at the start of each iteration and decreasing to a minimum of 30 s/m, before the sweating rate is adjusted. The maximum and minimum values of *r*_s_ were chosen based on the work of Webster (1974) and Turnpenny et al. (2000b).

### Heat loss from skin

#### Convective heat loss

Convective heat transfer between the outer surface of the haircoat and the ambient air can be written as:

A5 $$C=\frac{\rho {c}_{\mathrm{p}}}{r_{\mathrm{H}}}\left({T}_{\mathrm{c}}-{T}_{\mathrm{a}}\right)$$

Here, the convective resistance was estimated using the correlation by Wiersma and Nelson (1967), reproduced in Eq. (A6), with both the Reynolds number (Re) and Nusselt number (Nu) based on the trunk diameter, set in the present model to *d*_t_=0.5 m.

A6 $$\mathrm{Nu}=0.65\ {\operatorname{Re}}^{0.53}$$

#### Radiant heat exchange

Since heat stress in cattle sheltered in naturally ventilated barns is of main interest to the present study, the radiant exchange calculations were simplified by assuming no solar radiation (*S*_abs_=0) and considering only the long-wave radiant exchange with the surrounding surfaces. The net long-wave radiant exchange between the outer surface of the haircoat and the surroundings, *L*, can be written as shown in Eq. (A7) with the radiation resistance *r*_R_ obtained from the linearized Stefan’s Law.

A7 $$L=\frac{\rho {c}_{\mathrm{p}}}{r_{\mathrm{R}}}\left({T}_{\mathrm{c}}-{T}_{\mathrm{r}}\right)$$

#### Latent heat loss from skin

Heat loss through the evaporation of sweat on the skin (*E*_c_ [W/m^2^]) is a crucial heat dissipation mechanism, and its maximum a major determinant of the onset of heat stress. There is a physiological limit on the sweating rate, dictated by water availability and the activity of sweat glands (McArthur 1987). As pointed out by McArthur (1987) and Turnpenny et al. (2000b), *E*_c,max_ is normally determined by this physiological limit. Turnpenny et al. (2000b) used ${\hat{E}}_{\mathrm{c},\max }=120\ \mathrm{W}/{\mathrm{m}}^2$ as the physiological limit, derived from studies conducted in the 1970s. It is likely that the modern high-yield cow has a higher sweating capacity. A 2010 study (Gebremedhin et al. 2010), for instance, reports *E*_c,max_ as high as 280 W/m^2^ from cattle subject to hot and dry conditions in the shade, though the reported mean is less than half the maximum (138 W/m^2^). Maia et al. (2005a) have similarly reported measurements of *E*_c_ well exceeding 300 W/m^2^ for pasture-fed Holstein cows in a tropical climate, which is difficult to reconcile with typical metabolic heat generation rates (*M*<300 W/m^2^), especially for the relatively low-yielding cows (~15 kg/day) studied in that work. A 2011 study of Holstein cows (da Silva and Maia 2011), on the other hand, reports *E*_c_ < 100 W/m^2^. In the present work, unless otherwise specified, ${\hat{E}}_{\mathrm{c},\max }=120\ \mathrm{W}/{\mathrm{m}}^2$ was assumed, in accordance with Turnpenny et al. (2000b).

In high humidity, *E*_c_ may be supressed below the physiological limit due to low evaporation potential from the skin. As suggested in by Turnpenny et al. (2000b), this environmental limit can be estimated as:

A8 $${\overset{\sim }{E}}_{\mathrm{c},\max }=\frac{\rho {c}_{\mathrm{p}}}{\gamma}\left(\frac{e_{\mathrm{s}\mathrm{at}}\left({T}_{\mathrm{s}}\right)-{e}_{\mathrm{a}}}{r_{\mathrm{v}}}\right)$$

where *e* [Pa] is the vapor pressure, *γ* is the psychrometric constant (0.066 kPa/K), and *r*_v_ [s/m] is the lower limit of the resistance to mass transfer, namely the vapor transfer resistance due to diffusion and free convection through the haircoat, given by Cena and Monteith (1975) as:

A9 $${r}_{\mathrm{v}}=\frac{l}{D\left[1+1.54\frac{l}{d_t}{\left({T}_{\mathrm{s}}^{\ast }-{T}_{\mathrm{a}}^{\ast}\right)}^{0.7}\right]}$$

where *D* [m^2^/s] is the diffusivity of water vapor in air (2.5×10^−5^ m^2^/s at 20°C), and *T*_s_^*^ and *T*_a_^*^ [K] are the virtual temperature of skin and air, respectively. The virtual temperature is defined in Eq. (A10) (McArthur 1987).

A10 $${T}^{\ast }=\left(T+273.15\right)\left(1+0.38e/p\right)$$

To account for the effect of wind on the evaporation of sweat, *l* in Eq. (A9) is replaced with *l*–*l*_w_ where *l*_w_ is the “wind penetration depth” (Turnpenny et al. 2000a).

In general, *E*_c,max_ must be re-evaluated in each iteration as the smaller of the physiological and environmental limits;

A11 $${E}_{\mathrm{c},\max }=\min \left\{{\hat{E}}_{\mathrm{c},\max },{\overset{\sim }{E}}_{\mathrm{c},\max}\right\}$$

Under conditions of primary interest to the present study (*T*_a_ ≤ 40°C, RH<60%), ${\overset{\sim }{E}}_{\mathrm{c},\max }>120$W/m^2^ and the physiological limit of 120 W/m^2^ prevails.

As suggested by McArthur (1987), the evaporation of moisture from an animal’s body can take place below the skin surface. In cold, for instance, when the sweat glands are inactive and the skin surface is dry, there is water vapor loss by diffusion through the skin. Therefore, *E*_c,min_>0. According to McArthur (1987), *E*_c,min_ ≈ 0.04*E*_c,max_ in homeotherms. This baseline was implemented in the present model by initializing *E*_c_ at 0.04*E*_c,max_.

### Respiratory heat loss

Respiratory heat loss was calculated based on an energy balance between the inspired and expired air streams. The inspired air was assumed to be at the ambient conditions, i.e. re-inhalation of the expired air was ignored. The expired air was assumed to be saturated at a temperature calculated based on the empirical correlation proposed by Stevens (1981):

A12 $${T}_{\mathrm{ex}}=17+0.3{T}_a+\exp \left(0.01611\mathrm{RH}+0.0387{T}_{\mathrm{a}}\right)$$

As noted by Stevens (1981), the correlation above has two important implications:

1. The exhaled air is not at the body temperature, as assumed in many studies, e.g. McArthur (1987), Turnpenny et al. (2000a, b) and McGovern and Bruce (2000).
2. *T*_ex_ is a function of the ambient conditions only.

The respiration rate, *R* [br/min], and tidal volume, *V*_t_ [m^3^/br], were also calculated using correlations proposed by Stevens (1981):

A13 $$R=\exp \left(2.966+0.0218{T}_a+0.00069{T}_a^2\right)$$

A14 $${V}_{\mathrm{t}}=0.0189{R}^{-0.463}$$

Note the reverse trends of *V*_t_ and *R* as *T*_a_ increases, i.e. rapider but shallower respiration at higher *T*_a_. As noted by Stevens (1981), the inspired air cools the upper respiratory tract as it passes through. When air is expired, on the other hand, it is cooled by the respiratory passages. At a given *T*_a_, increasing RH reduces the cooling potential of the inhaled air. Therefore, the upper respiratory tract is cooled by the inhaled air to a lesser extent, leading to higher *T*_ex_.

The correlations above apply to first-phase panting only (Stevens 1981; Berman 2004). Furthermore, the correlations were obtained based on environmental chamber measurements with no induced air flow. Equation (A13) is therefore expected to overestimate *R* in open barns where there is virtually always air flow. An overestimated *R* will however only lead to a more conservative estimation of heat stress. Moreover, *E*_r_ is by far dominated by *E*_c_, making the overestimation of *E*_r_ even less problematic.

Alternatively, the comparable correlations proposed by Maia et al. (2005b) can be used to evaluate the respiratory heat loss as a function the ambient temperature only.

### Validation

In the absence of detailed heat-transfer measurements on the modern, high-yielding dairy cattle, data from the seminal work of Blaxter and Wainman (1964) on steers was used for validating the present implementation of the model by Turnpenny et al. (2000a,b). The measurements by Blaxter and Wainman (1964) were divided into seven groups of three trials based on the air temperature and speed. To simulate each group, the mean values were used to define the boundary conditions (*T*_a_, *u*) and animal characteristics (*m*, *l*, *M*, *T*_b_, *r*_s,min_) in the model. See Table 1. The ambient humidity and surface temperatures were not reported by Blaxter and Wainman (1964). In the model, a moderate humidity of RH*=*40% was assumed. Based on data from an earlier study using the same respiration chamber (Blaxter and Wainman 1962), it was assumed that *T*_r_=*T*_a_.

**Table A1:** Summary of experimental data from Blaxter and Wainman (1964) for steers, used in the validation study (μ: mean, σ: standard deviation, *E*: evpoartive heat loss)

Animal	*T*_a_ [°C]	u [m/s]	*l* [mm]	*m* [kg]	*E*_min_ [W/m_2_]	*r*_s,max_ [s/m]	*T*_b_ [°C]	*M* [W/m^2^]	*T*_s_ [°C]	*E* [W/m^2^]
J	18.9	0.72	22	359	16	186	38.3	89	30.8	40
I	18.6	0.72	23	343	15	224	38.6	87	32.8	40
G	19.3	0.72	19	357	20	194	38.2	87	30.2	36
μ	18.9	0.72	21	353	17	202	38.4	88	31.3	39
σ	0.4	0.00	2.1	8.7	3	20	0.2	1	1.4	2.5
I	19.0	0.72	6	324	15	224	38.1	91	28.3	26
J	19.4	0.72	12	348	16	186	37.9	95	29.1	24
G	19.5	0.72	11	341	20	194	38.3	97	29.9	28
μ	19.3	0.72	10	338	17	202	38.1	94	29.1	26
σ	0.3	0.00	3.2	12.3	3	20	0.2	3	0.8	1.8
I	17.9	0.18	23	343	15	224	38.7	87	32.6	41
G	18.3	0.18	18	345	20	194	38.5	93	33.4	39
J	18.8	0.18	22	362	16	186	38.2	88	32.3	40
μ	18.3	0.18	21	350	17	202	38.5	89	32.8	40
σ	0.5	0.00	2.6	10.4	3	20	0.3	3	0.6	0.8
G	0.3	0.18	24	358	20	194	38.4	97	24.4	21
J	−0.5	0.18	31	361	16	186	38.4	94	24.9	15
I	−1.1	0.18	29	337	15	224	38.6	88	23.3	15
μ	−0.4	0.18	28	352	17	202	38.5	93	24.2	17
σ	0.7	0.00	3.6	13.1	3	20	0.1	5	0.8	3.2
J	19.0	0.18	14	348	16	186	38.0	91	31.4	25
I	18.8	0.18	5	325	15	224	38.7	93	31.2	23
G	19.0	0.18	9	342	20	194	38.3	96	30.5	28
μ	18.9	0.18	9	338	17	202	38.3	93	31.0	25
σ	0.1	0.00	4.5	11.9	3	20	0.4	2	0.5	2.6
I	0.9	0.72	4	323	15	224	38.1	145	22.0	13
J	0.6	0.72	28	360	16	186	38.4	99	22.7	16
G	0.9	0.72	24	356	20	194	38.6	101	22.2	20
μ	0.8	0.72	19	346	17	202	38.4	115	22.3	17
σ	0.2	0.00	12.9	20.3	3	20	0.3	26	0.4	3.4
I	1.0	0.18	4	323	15	224	38.2	139	23.7	16
J	1.0	0.18	5	350	16	186	37.4	144	17.7	16
G	2.9	0.18	5	345	20	194	38.6	146	21.0	20
μ	1.6	0.18	5	339	17	202	38.1	143	20.8	17
σ	1.1	0.00	0.6	14.4	3	20	0.6	4	3.0	2.6

In Fig. A1, the model predictions for the skin temperature (*T*_s_) and total evaporative (latent) heat loss (*E*) are compared to the measurements (Blaxter and Wainman 1964). The horizontal error bars represent the two-standard-deviation band of the three trials in each group. The model predictions are in overall agreement with the measurements, particularly in the case of *T*_s_. In the absence of data that can be used for a more thorough validation, the comparison shown in Fig. A1 was taken as sufficient validation of the model.

## Footnotes

1 The empirical wind speed data used to derive the correction were in the range 0.6–15.5 m/s (Mader et al. 2010).

2 Note that indices defined in terms of *T* and RH, e.g. THI, can be recast in terms of *T*_db_ and *T*_wb_ (*c*_3_=0) using psychrometric relations or curve-fits.

3 *M* may initially increase due to panting but will eventually drop in response to prolonged heat exposure (McArthur 1987).

4 Blaxter and Wainman (1962) have similarly used the term “critical temperature” in assessing cold stress in steers to denote the temperature below which metabolism is increased to maintain the normal body temperature.

5 Mader et al. (2010) have similarly used “apparent temperature” as an alternative term for the Comprehensive Climate Index (CCI), defined as the dry-bulb temperature with three linear corrections for the effects of humidity, wind speed and radiation.

6 See the Appendix for more details about each limit.

## Nomenclature

*E*_c_: Cutaneous latent heat flux [W/m^2^]
${\hat{E}}_{\mathrm{c},\max }$: Physiological limit on cutaneous latent heat flux [W/m^2^]
*E*_r_: Respiratory heat flux [W/m^2^]
*G*_e_: Total heat dissipation flux [W/m^2^]
*M*: Metabolic heat generation flux [W/m^2^]
RH: Relative humidity [%]
*T*_a_: Ambient temperature [°C]
*T*_b_: Body temperature [°C]
*T*_ap_: Apparent temperature [°C]
${\overset{\sim }{T}}_{\mathrm{ap}}$: Apparent temperature for cattle [°C]
*T*_cr_: Critical temperature [°C]
*T*_s_: Skin temperature [°C]
TEI: Thermoregulatory exhaustion index [-]
THI: Temperature humidity index [-]
*u*: Air speed [m/s]
